# Supplementary material for: Tumors located in the brain impair the frequency and phenotype of dendritic cells in blood and tumor
Source: iScience. 2026 Feb 26;29(3):115160. doi: 10.1016/j.isci.2026.115160 (PMC12997301; doi:10.1016/j.isci.2026.115160)
Supplement: Document S1. Figures S1–S12 and Tables S1–S6 [file mmc1.pdf]

iScience, Volume *29*

Supplemental information

Tumors located in the brain impair the frequency and phenotype of dendritic cells in blood and tumor

Bryan Gardam, Tessa Gargett, Eunwoo Nam, Sidra Khan, Rebecca J. Ormsby, Santosh I. Poonnoose, Julie M. Bracken, Anupama Pasam, Sakthi Lenin, Briony L. Gliddon, Melinda N. Tea, Chloe L. Shard, Stuart M. Pitson, Guillermo A. Gomez, Katherine A. Pillman, Shahneen Sandhu, Michael P. Brown, and Lisa M. Ebert

(A)

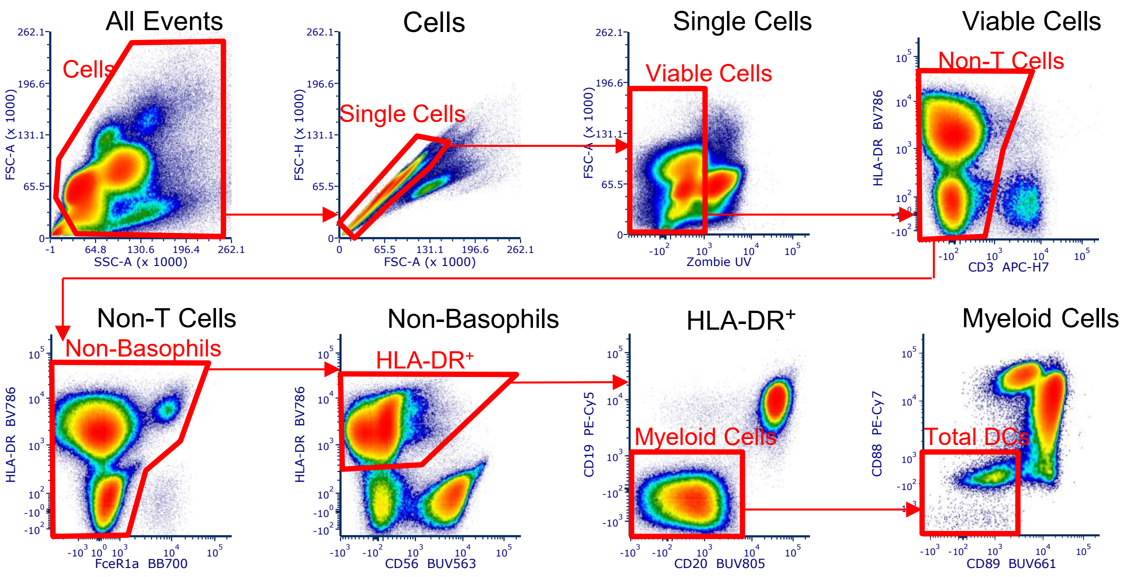

(B)

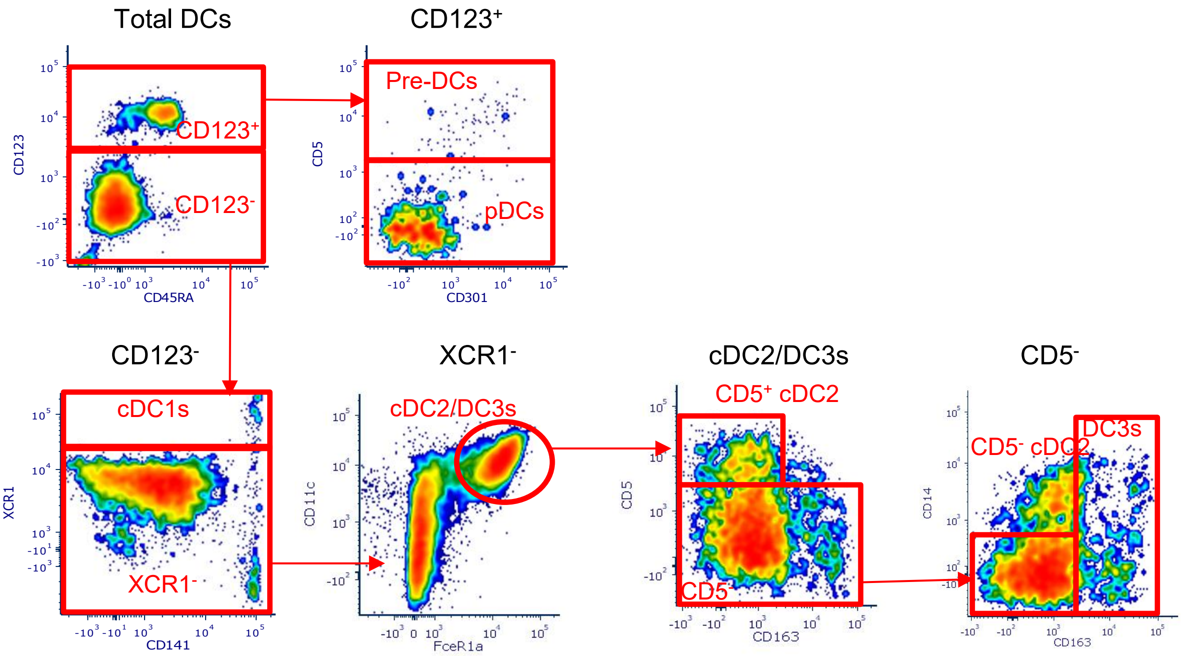

Figure S1. Indicative gating strategy of 21 colour flow cytometry panel to determine (A) the total dendritic cells, and (B) from total DCs to individual DC subsets adapted from Mair and Leichti (Mair & Liechti 2021). Related to Fig 1, 3, 5, and STAR Methods.

Table S1. Demographics of the healthy donor and patient groups utilized in this study. Related to Figure 1A

ID	Cohort	Gender (n=)	Median Age (range)	Brain Location	Steroids Before Surgery (time)
**Summary**	**Healthy Donors**	**Female 47% Male 53% (17)**	**53.94 (29-71)**		
HD1*	Healthy Donors	Female	44	N/A	N/A
HD1*	Healthy Donors	Female	47	N/A	N/A
HD2	Healthy Donors	Female	39	N/A	N/A
HD5	Healthy Donors	Female	29	N/A	N/A
HD9	Healthy Donors	Female	63	N/A	N/A
HD10	Healthy Donors	Female	56	N/A	N/A
HD12	Healthy Donors	Female	65	N/A	N/A
HD14	Healthy Donors	Female	70	N/A	N/A
HD3	Healthy Donors	Male	45	N/A	N/A
HD4	Healthy Donors	Male	35	N/A	N/A
HD6	Healthy Donors	Male	39	N/A	N/A
HD7	Healthy Donors	Male	48	N/A	N/A
HD8	Healthy Donors	Male	60	N/A	N/A
HD11	Healthy Donors	Male	71	N/A	N/A
HD13	Healthy Donors	Male	71	N/A	N/A
HD15	Healthy Donors	Male	67	N/A	N/A
HD16	Healthy Donors	Male	68	N/A	N/A
**Summary**	**Primary Glioblastoma**	**Female 43% Male 57% (14)**	**66.14 (46-75)**		
SANTB00501^	Primary Glioblastoma	Female	64	Right Occipital/Temporal	Yes (3 days)
SANTB00579	Primary Glioblastoma	Female	68	Left Occipital	Yes (9 days)
SANTB00651	Primary Glioblastoma	Female	75	Right Temporal	Yes (2 days)
SANTB00679	Primary Glioblastoma	Female	72	Right Parietal	Yes (6 days)
SANTB00713	Primary Glioblastoma	Female	76	Left Frontal	Yes (26 days)
SANTB00743-1	Primary Glioblastoma	Female	53	Left Frontal	Yes (6 days)
SANTB00502	Primary Glioblastoma	Male	67	Right Occipital	Yes (Long term)
SANTB00525	Primary Glioblastoma	Male	62	Right Frontal	Yes (5 days)
SANTB00598	Primary Glioblastoma	Male	75	Left Frontal	Yes (25 days)
SANTB00678	Primary Glioblastoma	Male	46	Right Parietal Inter-axial	Yes (7 days)
SANTB00683	Primary Glioblastoma	Male	69	Left Frontal	Yes (7 days)
SANTB00690	Primary Glioblastoma	Male	67	Left Temporal	Unk
SANTB00706	Primary Glioblastoma	Male	64	Left Temporal	Yes (1 day)
SANTB00707	Primary Glioblastoma	Male	68	Right Parietal	Yes (7 days)
**Summary**	**Recurrent Glioblastoma**	**Female 63% Male 37% (16)**	**52.25 (33-76)**		

SANTB00103	Recurrent Glioblastoma	Female	55	Right Temporal	Yes (1 day)
SANTB00118-2[#]	Recurrent Glioblastoma	Female	33	Left Temporal	Yes (Long term)
SANTB00118-3[#]	Recurrent Glioblastoma	Female	37	Left Temporal	Yes (3 days)
SANTB00501-2[^]	Recurrent Glioblastoma	Female	64	Right Occipital/Temproal	Yes (15 days)
SANTB00485-2[&]	Recurrent Glioblastoma	Female	51	Right Frontal	Yes (Long term)
SANTB00610	Recurrent Glioblastoma	Female	47	Left Frontal	No
SANTB00624	Recurrent Glioblastoma	Female	51	Left Occipital	Yes (1 day)
SANTB00544-2	Recurrent Glioblastoma	Female	55	Left Frontal	Yes (1 day)
SANTB00485-3[&]	Recurrent Glioblastoma	Female	53	Right Frontal	Yes (Long Term)
SANTB00486-2	Recurrent Glioblastoma	Female	76	Left Occipital	Yes (1 day)
CAREFOR-14	Recurrent Glioblastoma	Male	55	Right Temporal	Unk
CAREFOR-15	Recurrent Glioblastoma	Male	34	Right Temporal	Unk
SANTB00689	Recurrent Glioblastoma	Male	50	Left Temporal	Yes (2 days)
SANTB00663-2	Recurrent Glioblastoma	Male	59	Left Temporal	Yes (Long term)
SANTB00496-2	Recurrent Glioblastoma	Male	63	Left Parietal	Yes (1 day)
#210	Recurrent Glioblastoma	Male	53		No
**Summary**	**Low Grade Glioma**	**Female 43% Male 57% (7)**	**42.14 (32-61)**		
SANTB00154	Low Grade Glioma	Female	33	Left Frontal	Yes (1 day)
SANTB00682	Low Grade Glioma	Female	36	Right Frontal	Yes (6 days)
SANTB00702	Low Grade Glioma	Female	34	Right Occipital	Yes (9 days)
SANTB00150	Low Grade Glioma	Male	43	Right Frontal	Yes (32 days)
SANTB00215	Low Grade Glioma	Male	32	Right Frontal Temporal	Yes (2 days)
SANTB00687	Low Grade Glioma	Male	56	Right Frontal	No
SANTB00694	Low Grade Glioma	Male	61	Left Frontal	Yes (11 days)
**Summary**	**Brain Metastasis**	**Female 23% Male 77% (22)**	**57.32 (30-80)**		
SANTB00555	Brain Metastasis	Female	80	Right Occipital	Yes (1 day)
SANTB00260	Brain Metastasis	Male	79	Right Parietal	Yes (9 days)
SANTB00503	Brain Metastasis	Male	30	Right Frontal	Yes (Long Term)
SANTB00603	Brain Metastasis	Male	51	Right Parietal	Yes (1 day)
SANTB00655	Brain Metastasis	Male	59	Left Parietal	Yes (Long Term)
SANTB00688	Brain Metastasis	Male	73	Left Frontal	Yes (7 days)
SANTB00614	Brain Metastasis	Male	69	Right Parietal	Yes (8 days)
SANTB00625	Brain Metastasis	Male	81	Left Frontal	No

SANTB00692	Brain Metastasis	Male	61	Right Frontal	Unk
SANTB00693	Brain Metastasis	Male	70	Left Frontal	Yes (3 days)
SANTB00725-1	Brain Metastasis	Male	79	Right Frontal	Yes (2 days)
#303	Brain Metastasis	Male	45	Right Frontal	No
#303(2)	Brain Metastasis	Male	49	Right Frontal	No
#304	Brain Metastasis	Male	40	Left Parietal and Left Occipital	No
PMC0081-1	Brain Metastasis	Female	45	Left Frontal	No
BIO-005	Brain Metastasis	Female	45	Left Temporal	No
BIO-091	Brain Metastasis	Female	39	Posterior Lobe	No
PMC0081-2	Brain Metastasis	Female	45	Left Frontal	No
BIO-120	Brain Metastasis	Male	53	Left Cerebellar	No
BIO-140	Brain Metastasis	Male	56	Left Frontal	No
BIO-222	Brain Metastasis	Male	50	Right Temporal	No
CHA020	Brain Metastasis	Male	62	Left occipital	No
**Summary**	**Type of Primary Cancer**	**Female 59% Male 41% (17)**	**66.88 (50-84)**		
Non-brain tumor 1	Melanoma	Female	75	None	No
Non-brain tumor 2	Melanoma	Female	58	None	No
Non-brain tumor 4	Melanoma	Female	50	None	No
Non-brain tumor 6	Melanoma	Female	77	None	No
Non-brain tumor 7	Lung Cancer	Female	66	None	Yes (Long Term)
Non-brain tumor 8	Lung Cancer	Female	62	None	Yes (Long Term)
Non-brain tumor 9	Lung Cancer	Female	71	None	No
Non-brain tumor 11	Lung Cancer	Female	62	None	No
Non-brain tumor 12	Lung Cancer	Female	69	None	No
Non-brain tumor 13	Melanoma	Female	73	None	Yes (9 days)
Non-brain tumor 3	Melanoma	Male	66	None	No
Non-brain tumor 5	Melanoma	Male	78	None	No
Non-brain tumor 10	Lung Cancer	Male	80	None	No
Non-brain tumor 14	Melanoma	Male	81	None	Yes (4 days)
Non-brain tumor 15	Melanoma	Male	74	None	Yes (Long Term)
Non-brain tumor 16	Melanoma	Male	84	None	Yes (Long Term)
Non-brain tumor 17	Melanoma	Male	71	None	No

Notes:
* Two individual donations
#, ^,& Are the same patients
Steroids Before Surgery Yes (1 day) is for the day of surgery only

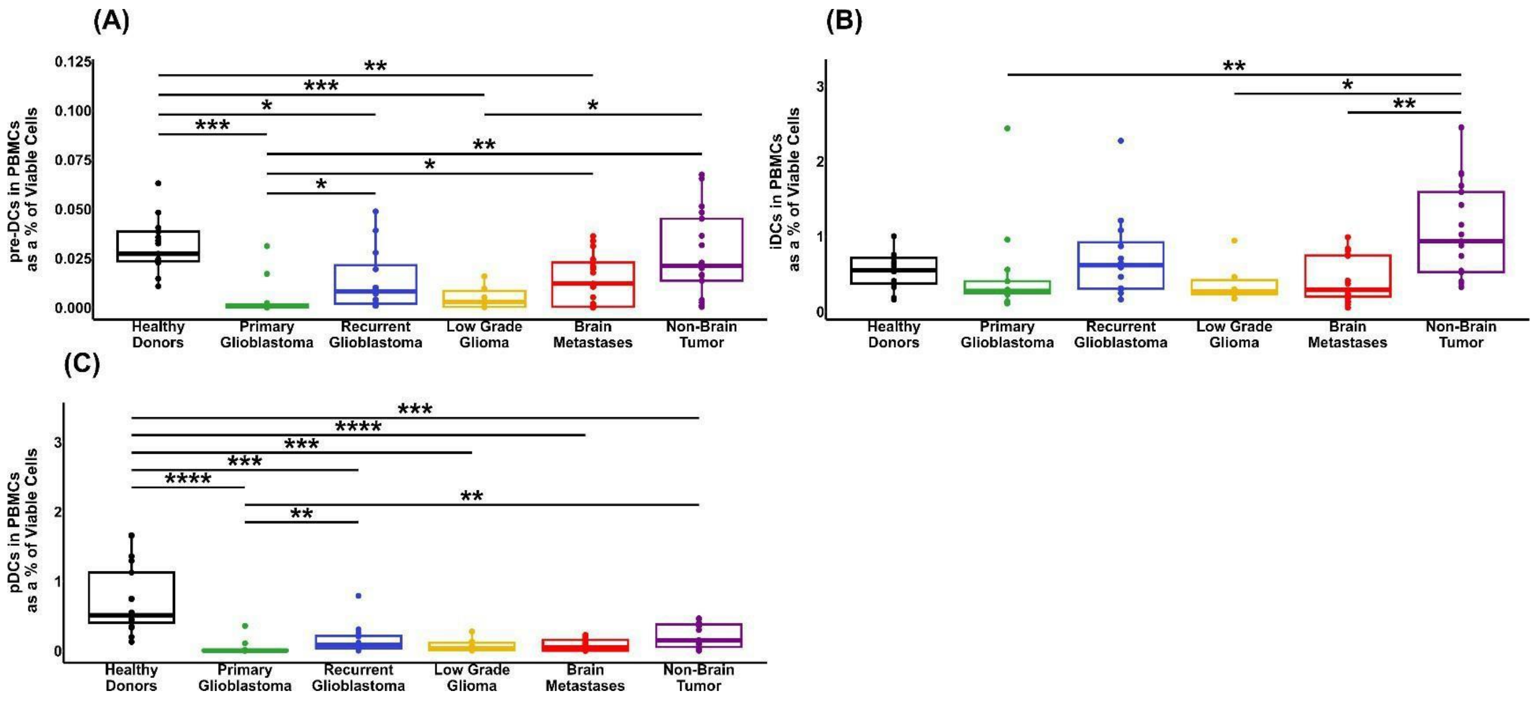

Figure S2. Quantification of the percentages of DC subsets as a percentage of the total viable cells from PBMCs. (A) pre-DCs (B) iDCs (C) pDCs. P values *p<0.05, **P<0.01, ***p<0.001, ****p<0.0001 Related to Figure 1

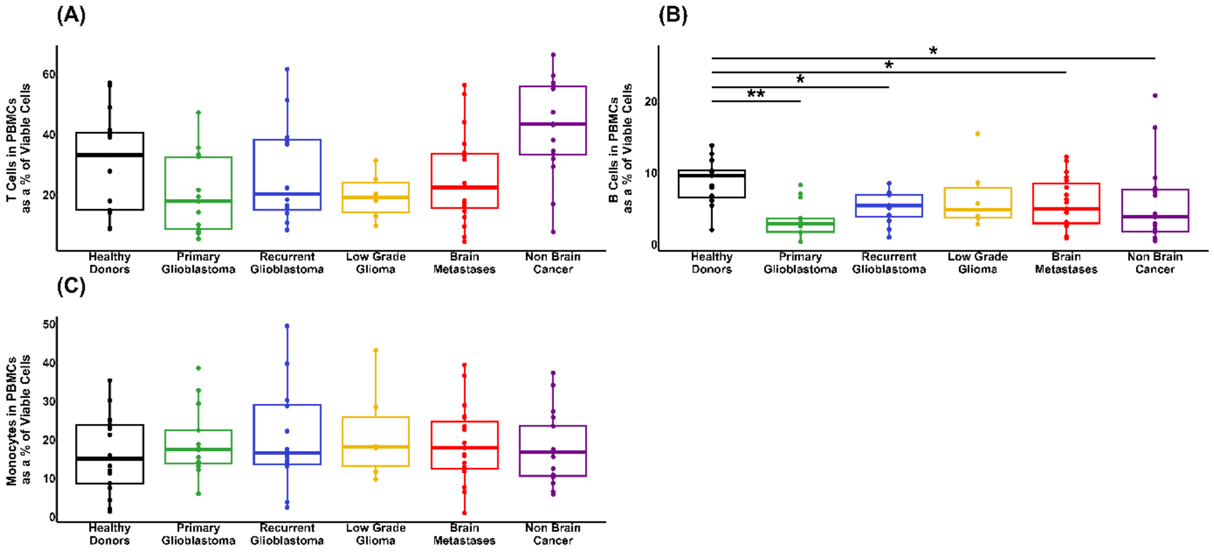

Figure S3. Proportion of cells across the patient groups shown as a percentage of viable cells (A) T cells, (B) B cells, and (C) Monocytes. P values *p<0.05, **P<0.01, ***p<0.001, ****p<0.0001. Related to Figure 1

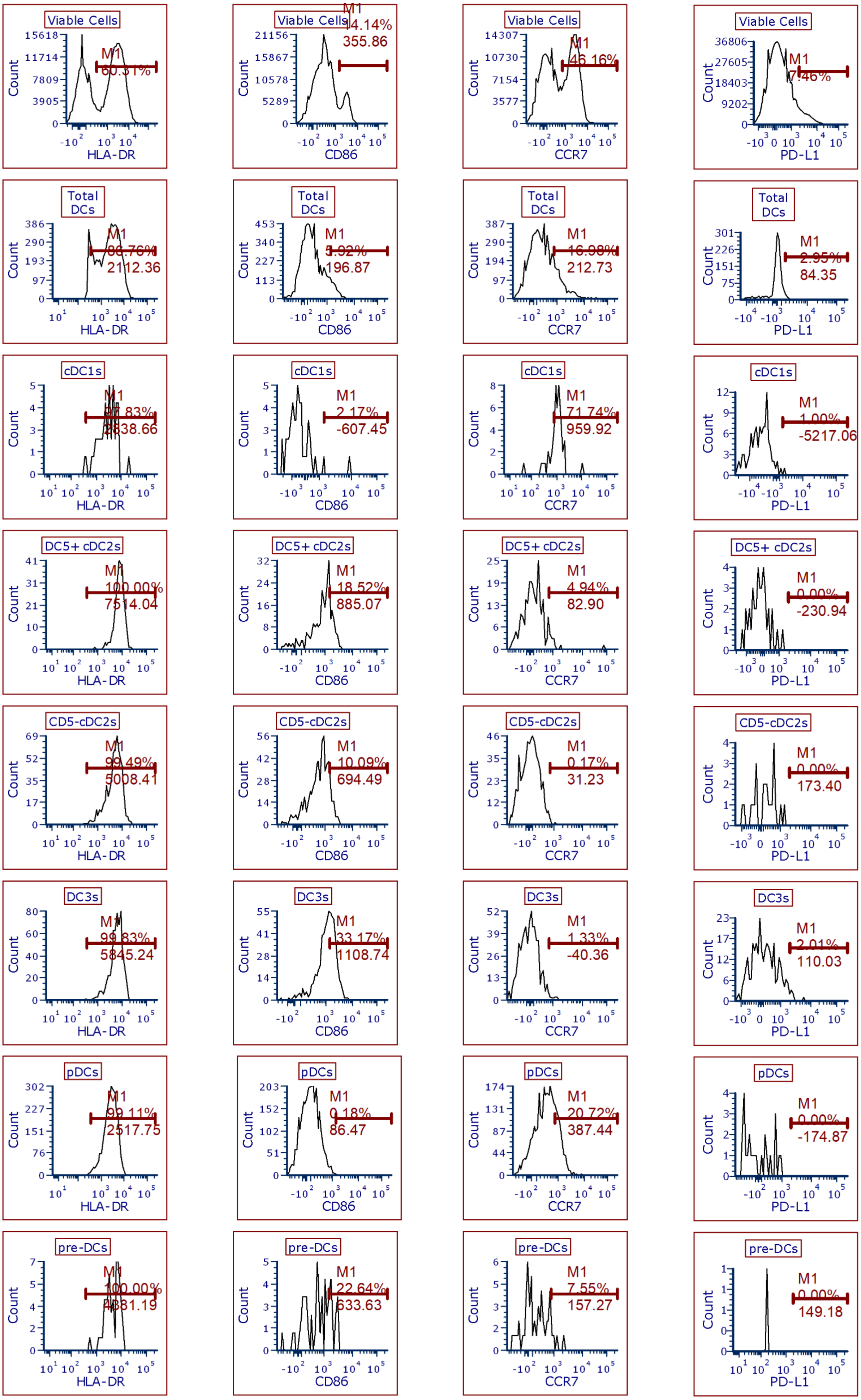

Figure S4. Indicative strategy for the calculation of MFI in human DC subsets. Related to Figure 3

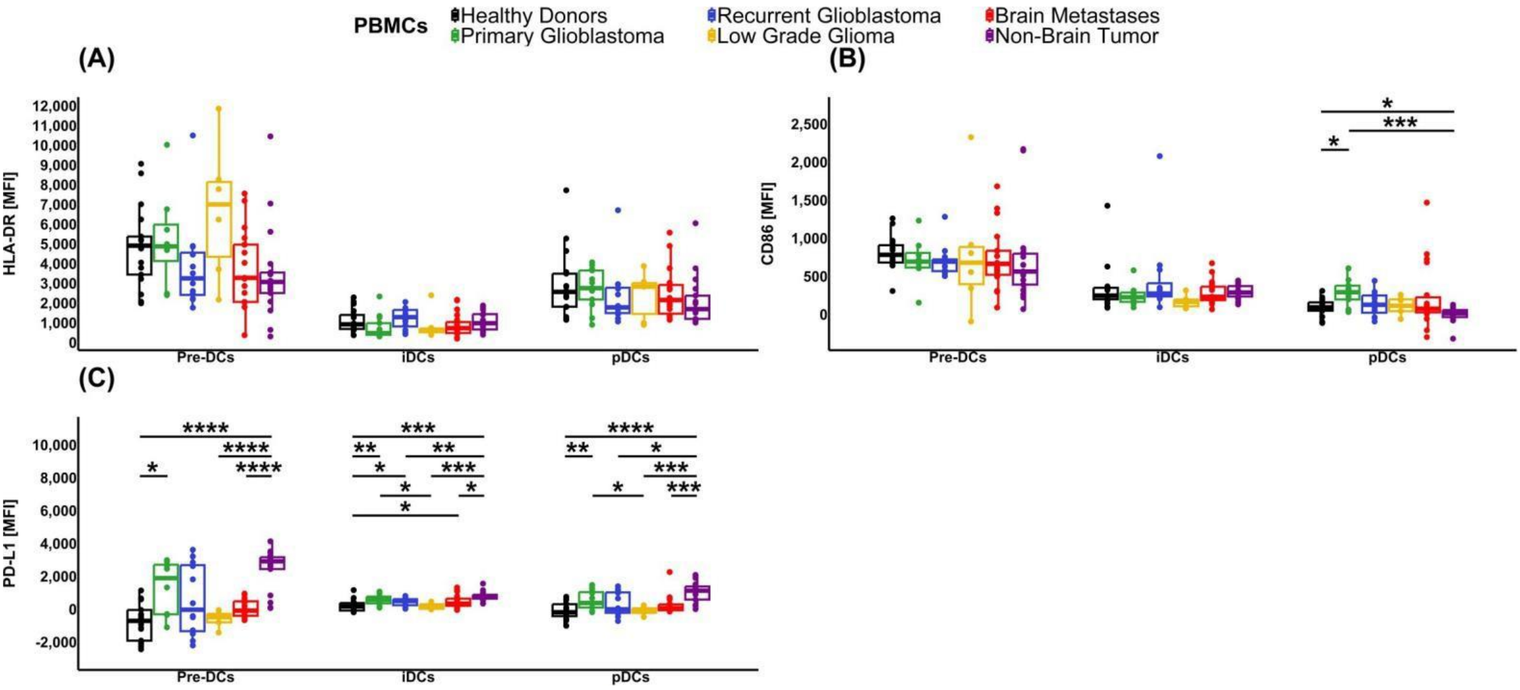

Figure S5 Quantification of pre-DCs, iDCs, and pDCs from PBMCs for (A) the MFI of HLA-DR. (B) the MFI of CD86, and (C) the MFI of PD-L1.

P values *p<0.05, **P<0.01, ***p<0.001, ****p<0.0001. Related to Figure 3

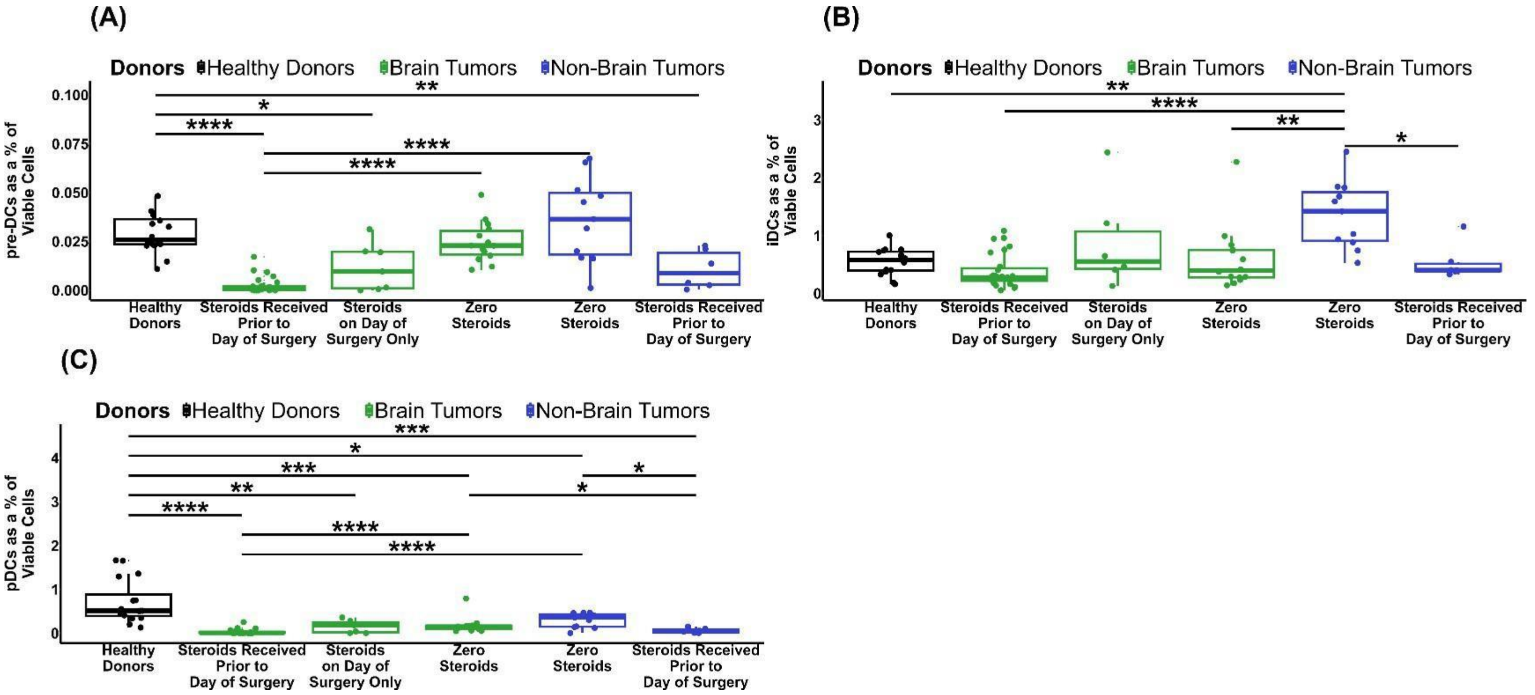

Figure S6. Steroid usage in combined brain tumour patients and non-brain tumour patients in (A) pre-DCs, (B) iDCs, and (C) pDCs.

P values *p<0.05, **P<0.01, ***p<0.001, ****p<0.0001. Related to Figure 4

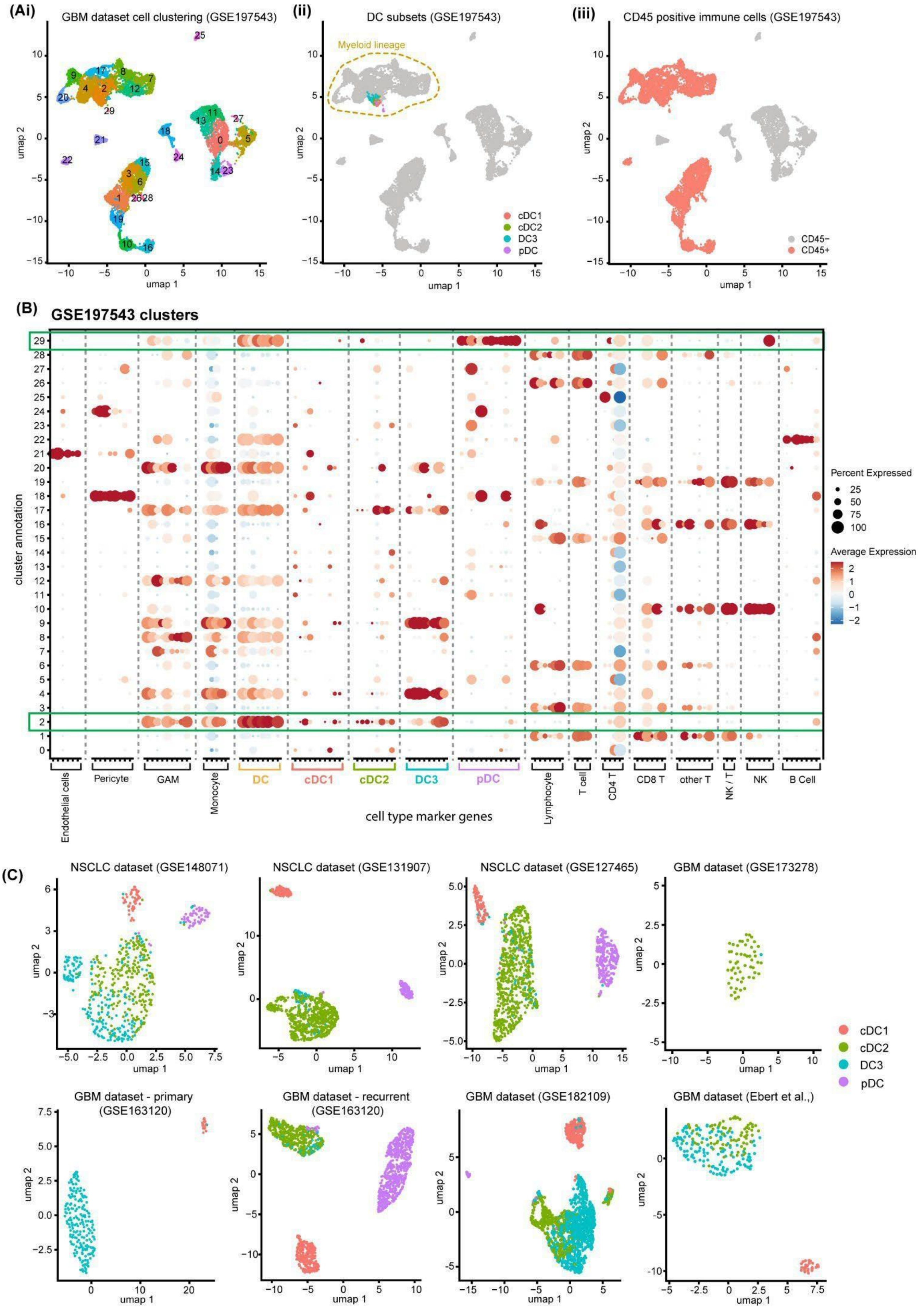

Figure S7 Detailed information of sub-setting of scRNA-seq datasets (A) UMAP plots illustrating (i) total cell population clustering, (ii) localisation of dendritic cells (DCs) within the myeloid lineage cluster, and (iii) annotation of CD45^{+} immune cells in a GBM scRNA-seq dataset (GSE197543). (B) Expression of cell type markers across cell clusters in the GBM dataset (GSE197543); green boxes indicate enrichment of DC subset marker genes in clusters 2 and 29. (C) UMAP plots showing DC subsets in all NSCLC and GBM datasets (datasets GSE197543 and GSE136246 shown in Figure 5). Related to Figure 5

Table S2. List of broad cell type and DC subset-specific marker genes utilized in the scRNA-seq analysis Related to Figure 5 and STAR Methods

Cell Type	Markers			
Endothelial cells	VWF	CLDN5	CD34	
	APOLD1	TIE1	CDH5	
Pericyte	PDGFRB	COL4A2	COL4A1	ACTA2
	MCAM	CSPG4	FN1	THY1
Glioma Accosiated Macrphage	AIF1	CSF1R	ITGAX	TREM2
	FCER1G	GPR34	LAPTM5	CD83
	RGS10			
Lymphocyte	CD2	CD6	IL7R	CD96
	CD247	LTB		
Monocyte/Macrophage	C5AR1			
Dendritic Cell	CD74	HLA-DRB1	HLA-DQA1	CST3
	HLA-DPA1	HLA-DPB1	HLA-DRA	HLA-DQB1
cDC1	CADM1	CLNK	C1orf54	RAB32
	XCR1	WFDC21P	IDO1	BATF3
	CLEC9A	DNASE1L3	LGALS2	FLT3
cDC2	CD1C	CD1E	CD86	RALA
	FCER1A	CLEC4A	C15orf48	PKIB
	CLEC10A	RALA		
DC3	VCAN	S100A9	CD36	LYZ
	S100A8	FCN1	CD163	CD14
pDC	IL3RA	ASIP	TPM2	SPIB
	BCL11A	PTCRA	MZB1	ITM2C
	TCF4	LRRC26	SMPD3	SERPINF1
	LILRA4			
Monocyte	CTSS	NEAT1	PSAP	SERPINA1
TCells	CD3D	CD3G	TRAC	
CD4 + T Cells	MAL	CD4	LDHB	TPT1
CD8+ Tcells	CD8B	CD8A	HCST	CD3E
	LINC02446	CTSW		
Other TCells	TRDC	GZMK	KLRB1	GZMA
	TRGC2	LYAR	KLRG1	
NK TCells	NKG7	CST7		
NK Cells	KLRD1	GNLY	PRF1	GZMB
	KLRF1			
B Cells	CD791	RALGPS2	CD79B	IGHM
	MS4A1	BANK1	TNFRSF13C	MEF2C

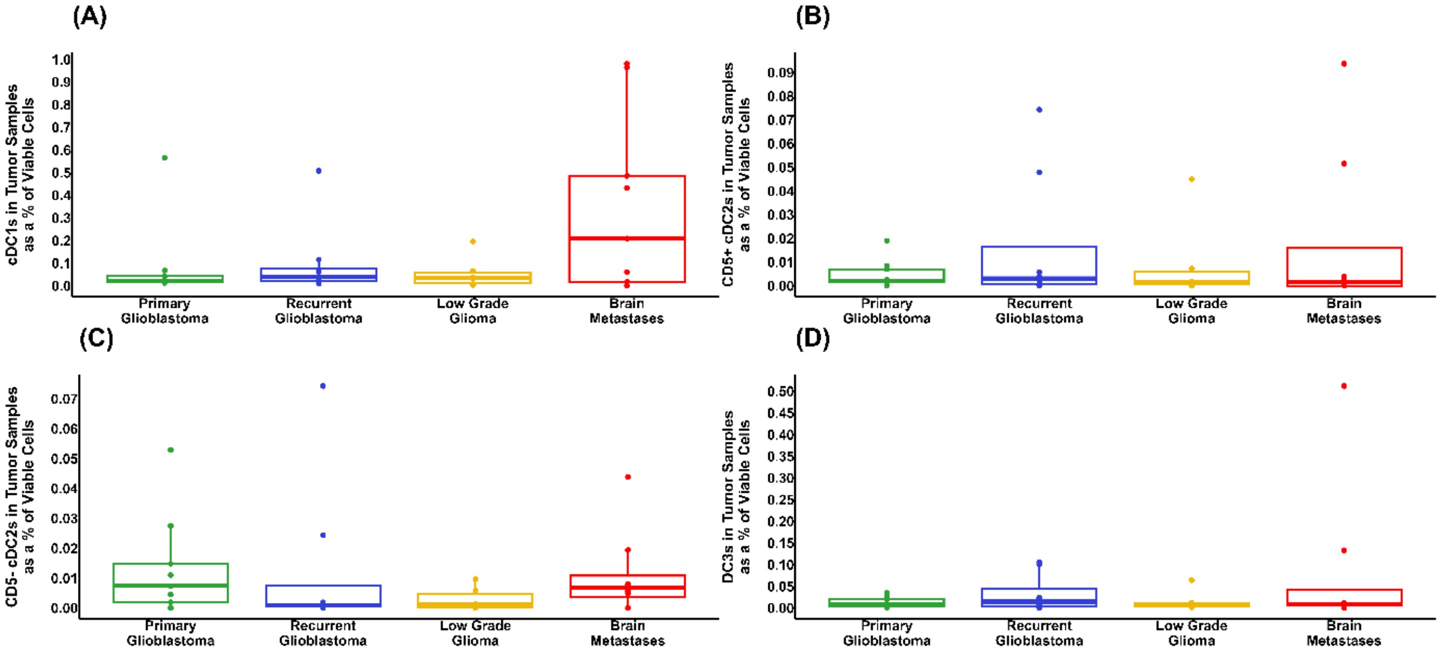

Figure S8. Quantification of DC subsets identified by flow cytometry in patient tumor samples (A) cDC1s, (B) CD5+ cDC2s, (C) CD5- cDC2s, and (D) DC3s. Related to Figure 5

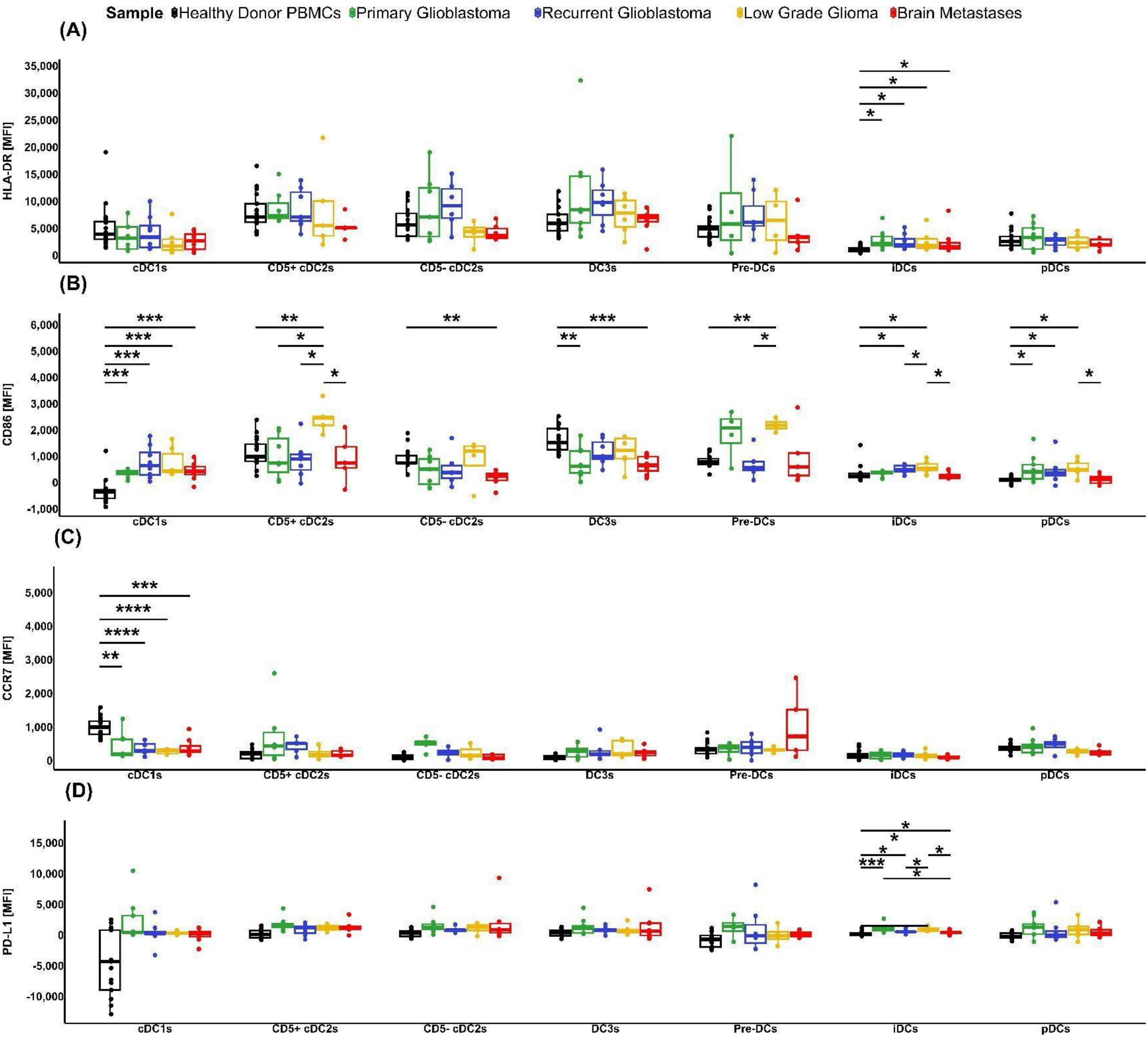

Figure S9. Quantification of DC subsets in patient tumor samples compared to healthy donor PBMCs for (A) the MFI of HLA-DR. (B) the MFI of CD86, (C) MFI of CCR7 and (D) the MFI of PD-L1. P values *p<0.05, **P<0.01, ***p<0.001, ****p<0.0001. Related to Figure 5.

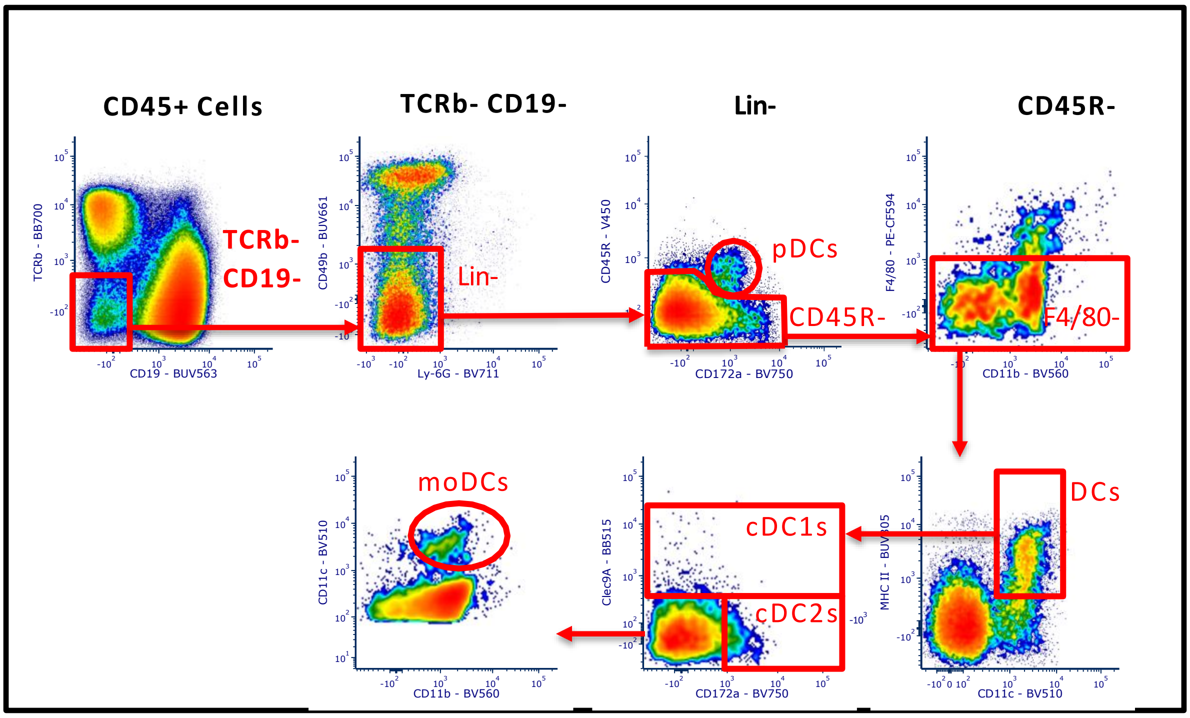

Figure S10. Indicative gating strategy for C57BL/6 mouse to identify the murine dendritic cells and subsets. Related to Figure 6 and STAR Methods.

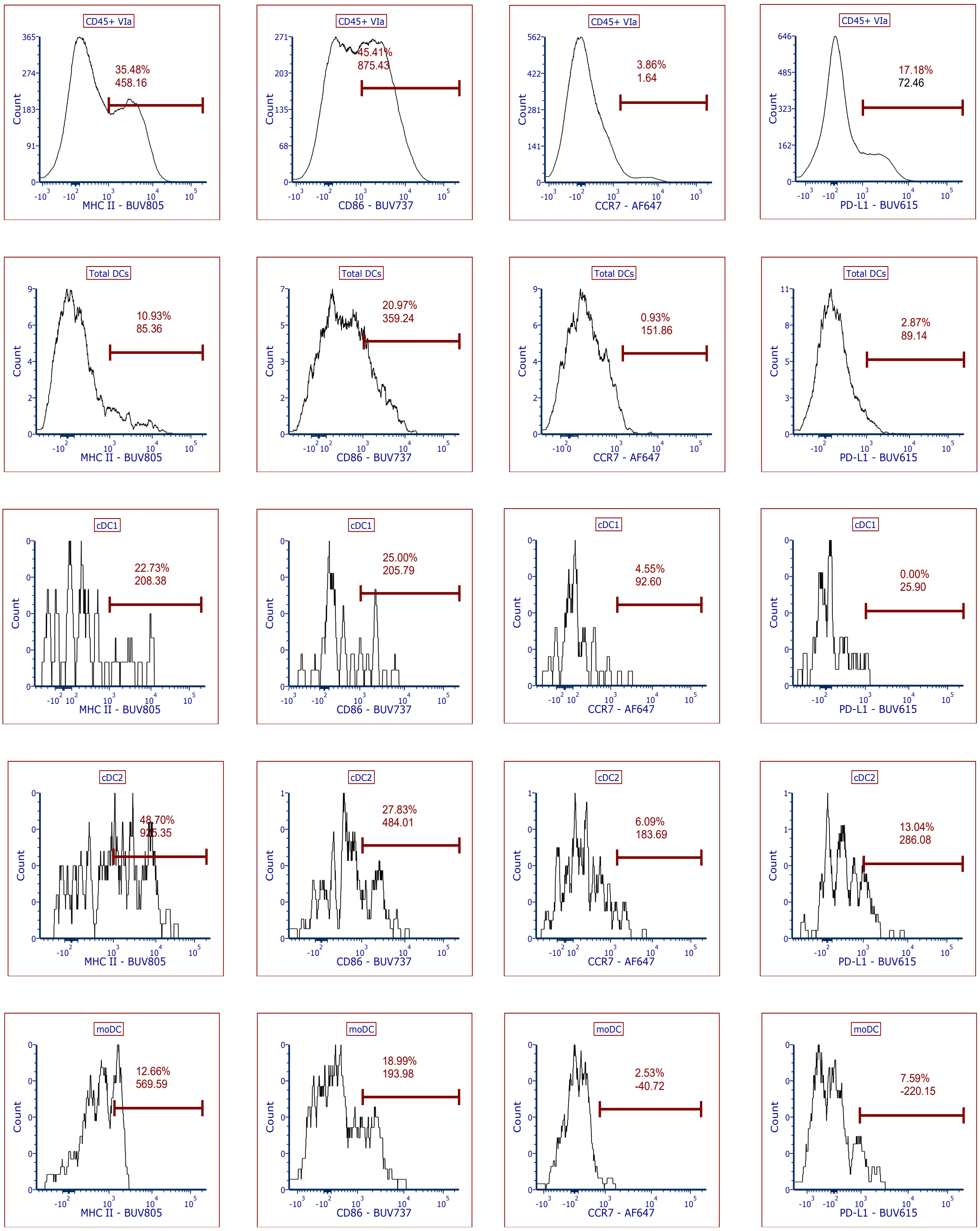

Figure S11. Indicative strategy for the calculation of MFI in mouse DC subsets. Related to Figure 6.

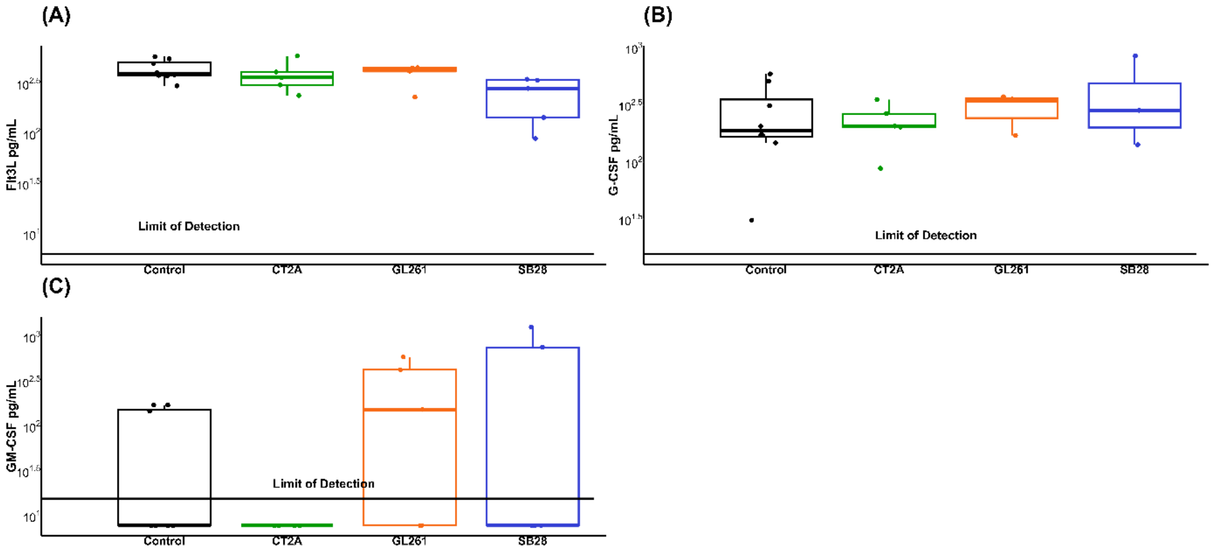

Figure S12. Quantification of DC growth factors in mouse serum (A) FLT3L, (B) G-CSF, and (C) GM-CSF. Results below the limit of detection (LOD) were calculated as half of the lower LOD as described in Giskeødegård & Lydersen[58] Related to Figure 6.

Table S3 . Corticosteroid usage data for patients with brain tumors. Related to STAR Methods

ID	Gender	Age	Pathological diagnosis	Date of surgery	Steroid date commenced	Steroid	Steroid dosage
SANTB00118-2	Female	33	WHO Grade IV	16/10/2017	Pre-admission	1mg Dexamethasone	4mg x 1 times day for surgery
SANTB00118-3	Female	37	WHO Grade IV	5/05/2021	3/05/2021	Nil pre-admission	8mg x 2 times for surgery
SANTB00150	Male	43	Grade III anaplastic astrocytoma	11/10/2017	28/09/2017	2mg Dexamethasone	4mg x 3 times a day on 6/10/17
SANTB00154	Female	33	Grade III anaplastic oligodendroglioma	25/10/2017	25/10/2017	Nil pre-admission	4mg x 4 times a day for surgery
SANTB00215	Male	32	High-grade astrocytoma	25/07/2018	23/07/2018	Nil pre-admission	4mg x 4 times a day for surgery
SANTB00260	Male	79	Metastatic squamous cell carcinoma	30/01/2019	22/01/2019	Nil pre-admission	4mg x 4 times a day for surgery
SANTB00485-2	Female	51	WHO Grade IV	8/11/2021	1/11/2020	0.5mg x 1 long-term Dexamethasone	4mg x 3 for surgery
SANTB00485-3	Female	53	WHO Grade IV	22/05/2023	pre admission	0.5mg x 1 long-term Dexamethasone	0.5mg x 1 times a day for surgery
SANTB00496-2	Male	63	WHO Grade IV	5/04/2023	5/04/2023	Nil pre-admission	4mg x 3 times a day for surgery
SANTB00501	Female	64	WHO Grade IV	16/12/2020	14/12/2020	Nil pre-admission	8mg x 2 times on day of surgery
SANTB00501-2	Female	64	WHO Grade IV	8/05/2021	24/03/2021	unsure	unsure
SANTB00502	Male	67	WHO Grade IV	4/01/2021	pre admission	2mg Dexamethasone continuing	8mg x 2 times on day surgery
SANTB00503	Male	30	Metastatic sarcoma	4/01/2021	pre admission	2mg Dexamethasone	8mg x 2 times on day surgery
SANTB00525	Male	62	WHO Grade IV	25/03/2021	21/03/2021	Nil pre-admission	8mg x 2 times on day surgery
SANTB00546	Female	41	WHO Grade IV	28/04/2021	24/04/2021	Nil pre-admission	8mg x 2 times for surgery
SANTB00555	Female	80	Metastatic carcinoma	18/05/2021	18/05/2021	Nil pre-admission	4mg x 4 times day on 18/5/21
SANTB00579	Female	68	WHO Grade IV	13/08/2021	5/08/2021	Dexamethasone	4mg x 4 times a day for surgery
SANTB00598	Male	75	WHO Grade IV	22/10/2021	28/09/2021	Dexamethasone	4mg x 4 times a day for surgery
SANTB00603	Male	51	High-grade B-cell lymphoma	3/11/2021	surgery only	Nil pre-admission	4mg x 4 times a day for surgery
SANTB00610	Female	47	WHO Grade IV	24/11/2021	nil	nil	nil
SANTB00624	Female	51	WHO Grade IV	21/03/2022	surgery only	nil	4mg x 4 times a day for surgery
SANTB00651	Female	75	WHO Grade IV	18/07/2022	16/06/2022	nil	4mg x 4 times a day for surgery
SANTB00663-2	Male	59	WHO Grade IV	21/03/2023	pre admission	0.25mg x1 long-term Dexamethasone	4mg x 4 times day for surgery
SANTB00679	Female	72	WHO Grade IV	9/01/2023	4/01/2023	nil pre-admission	4mg x 3 times a day for surgery
SANTB00682	Female	36	Oligodendroglioma WHO Grade II	24/01/2023	19/01/2023	Nil pre-admission	8mg x 2 times per day for surgery

SANTB00683	Male	69	WHO Grade IV	31/01/2023	24/01/2023	nil pre-admission	4mg x 3 times a day for surgery
SANTB00687	Male	56	Oligodendroglioma WHO Grade II	28/02/2023	nil	nil pre-admission	nil
SANTB00688	Male	73	Metastatic melanoma	7/03/2023	1/03/2023	nil pre-admission	4mg x 4 times day for surgery
SANTB00689	Male	50	WHO Grade IV	8/03/2023	7/03/2023	nil pre-admission	8mg x 2 times per day for surgery
SANTB00692	Male	61	Metastatic clear cell renal cell carcinoma	16/03/2023	unsure	nil pre-admission	4mg x 3 times a day for surgery
SANTB00693	Male	70	Metastatic poorly differentiated carcinoma	20/03/2023	23/03/2023	nil pre-admission	4mg x 3 times a day for surgery
SANTB00694	Male	61	Anaplastic oligodendroglioma	29/03/2023	19/03/2023	Nil pre-admission	4mg x 2 times a day for surgery
SANTB00706	Male	64	WHO Grade IV	9/05/2023	9/05/2023	Nil pre-admission	4mg x 4 times day for surgery
SANTB00614	Male	69	Metastatic melanoma	12/01/2022	5/01/2022	Nil pre-admission	8mg BD
SANTB00707	Male	68	WHO Grade IV	19/05/2023	13/05/2023	4mg x 4 per day Dexamethasone	4mg x 4 per day for surgery
SANTB00713	Female	76	WHO Grade IV	5/06/2023	10/5/23 (4 days) 4mg 2x day to wean	nil on admission	4mg x 4 per day for surgery
SANTB00486-2	Female	76	WHO Grade IV	31/07/2023	31/07/2023	nil on admission	4mg x 2 per day for surgery
SANTB00743-1	Female	53	WHO Grade IV	12/09/2023	6/09/2023	nil pre-admission	4mg x 4 per day for surgery
NonCNS 1	Female	75	Metastatic melanoma	25/05/2021		nil pre-admission	
NonCNS 2	Female	53	Metastatic melanoma	30/07/2021		nil pre-admission	
NonCNS 3	Male	66	Metastatic melanoma cervical LN met	19/08/2022		nil pre-admission	
NonCNS 4	Female	50	Metastatic melanoma	Jul-21		prednisolone	10mg daily
NonCNS 5	Male	78	Metastatic melanoma	Feb-21		nil pre-admission	
NonCNS 6	Male	77	Metastatic melanoma	Not specified		nil pre-admission	
NonCNS 7	Female	66	Recurrent NSCLC	Not specified		Prednisolone	37.5mg-10mg daily
NonCNS 8	Female	62	Metastatic NSCLC	Not specified	18/12/2023	beclometasone - 100microgram-6microgram-10microgram	Twice daily
NonCNS 9	Female	71	Metastatic NSCLC	Not specified		dexamethasone	dexamethasone 2mg daily
NonCNS 10	Male	80	NSCLC	Not specified		nil pre-admission	
NonCNS 11	Female	62	Metastatic NSCLC	Not specified		nil pre-admission	
NonCNS 12	Female	69	Metastatic NSCLC	15/03/2023		prednisolone	5mg daily
NonCNS 13	Female	73	Metastatic melanoma	Not specified		dexamethasone	4mg twice daily
NonCNS 14	Male	81	Metastatic melanoma	Not specified		prednisolone	5mg daily

NonCNS 15	Male	74	Metastatic melanoma	Not specified		methylprednisolone	2mg/kg
NonCNS 16	Male	84	Metastatic melanoma	Not specified		prednisolone	not specified
NonCNS 17	Male	71	Metastatic melanoma	Not specified		dexamethasone	8mg daily

Table S4. Antibodies that were utilized for staining human PBMCs and tumour samples for the identification of DC and subsets. Related to STAR Methods.

Antibody	Conjugate	Clone	Supplier	Catalogue #
Viability	Zombie UV		BioLedgend	423107
CD3	APC-H7	SK7	BD Biosciences	560176
FcεR1α	BB700	AER-37	BD Biosciences	747780
CD56	BUV563	NCAM16.2	BD Biosciences	612928
CD19	PE-Cy5	HIB19	BD Biosciences	555414
CD88	PE-Cy7	S5/1	BioLedgend	344308
CD89	BUV661	A59	BD Biosciences	750616
IL-3Rα (CD123)	BB515	6H6	BD Biosciences	567715
CD45RA	AF700	HI100	BD Biosciences	560673
CD141	BUV615	1A4	BD Biosciences	752356
XCR1	BV421	S15064E	BioLedgend	372610
CD5	BV480	UCHT2	BD Biosciences	566122
CD301 (CLEC10A)	PE	H037G3	BioLedend	354704
CD11c	APC	B-ly6	BD Biosciences	559877
CD163	BUV737	GHI/61	BD Biosciences	741863
CD14	BUV395	MφP9	BD Biosciences	563561
HLA-DR	BV786	G46-6	BD Biosciences	564041
CD86	BV650	BU63	BD Biosciences	747528
CD274 (PD-L1)	PE-CF594	MIH1	BD Biosciences	563742
CCR7 (CD197)	BV711	2-L1-A	BD Biosciences	566752

Table S5. Antibodies that were utilized for staining murine tissue samples for the identification of DC and subsets. Related to STAR Methods

Antibody	Conjugate	Clone	Supplier	Catalogue #
Viability	FVS 780		BD Biosciences	565388
CD45	BUV395	30-F11	BD Biosciences	564279
FcεR1α	BUV496	MAR-1	BD Biosciences	751763
CD172a	BV750	P84	BD Biosciences	747007
CD11b	BV650	M1/70	BD Biosciences	563402
CD19	BUV563	1D3	BD Biosciences	749028
TCR b	BB700	H57-597	BD Biosciences	745846
CD49b	BUV661	HMα2	BD Biosciences	741523
Ly-6G	BV711	1A8	BD Biosciences	563979
CD45R	V450	MAR-1	BD Biosciences	751763
F4/80	PE-CF594	T45-2342	BD Biosciences	565613
I-A/I-E (MHC II)	BUV805	2G9	BD Biosciences	748707
CD11c	BV510	N418	BD Biosciences	744178
Clec9A (CD370)	BB515	10B4	BD Biosciences	565320
Ly-6C	PE-Cy7	AL-21	BD Biosciences	560593
Siglec-F	APC-R700	E50-2440	BD Biosciences	565183
CD115	BV605	T38-320	BD Biosciences	743640
PD-L1 (CD274)	BUV615	MIH5	BD Biosciences	752339
CCR7 (CD197)	AF647	4B12	BD Biosciences	560766
CD86	BUV737	PO3	BD Biosciences	741757
CD8a	PE	5H10-1	BD Biosciences	567630

Table S6. Packages utilized in RStudio. Related to STAR Methods

**Package**	**Version**	**Reference**
tidyr	1.3.1	Wickham H, Vaughan D, Girlich M (2024). *tidyr: Tidy Messy Data*. R package version 1.3.1, https://github.com/tidyverse/tidyr, https://tidyr.tidyverse.org.
ggplot2	3.5.2	H. Wickham. ggplot2: Elegant Graphics for Data Analysis. Springer-Verlag New York, 2016.
dplyr	1.1.4	Wickham H, François R, Henry L, Müller K, Vaughan D (2023). *dplyr: A Grammar of Data Manipulation*. R package version 1.1.4, https://github.com/tidyverse/dplyr, https://dplyr.tidyverse.org.
ggpubr	0.6.0	Kassambara A (2023). *ggpubr: 'ggplot2' Based Publication Ready Plots*. R package version 0.6.0, https://rpkgs.datanovia.com/ggpubr/.
ggrepel	0.9.6	Slowikowski K (2024). *ggrepel: Automatically Position Non-Overlapping Text Labels with 'ggplot2'*. https://ggrepel.slowkow.com/, https://github.com/slowkow/ggrepel.
stringr	1.5.1	Wickham H (2023). *stringr: Simple, Consistent Wrappers for Common String Operations*. R package version 1.5.1, https://github.com/tidyverse/stringr, https://stringr.tidyverse.org.
rstatix	0.7.2	Kassambara A (2023). *rstatix: Pipe-Friendly Framework for Basic Statistical Tests*. R package version 0.7.2, https://rpkgs.datanovia.com/rstatix/.
